# Stable Sparse Classifiers Identify qEEG Signatures that Predict Learning Disabilities (NOS) Severity

**DOI:** 10.3389/fnins.2017.00749

**Published:** 2018-01-15

**Authors:** Jorge Bosch-Bayard, Lídice Galán-García, Thalia Fernandez, Rolando B. Lirio, Maria L. Bringas-Vega, Milene Roca-Stappung, Josefina Ricardo-Garcell, Thalía Harmony, Pedro A. Valdes-Sosa

**Affiliations:** ^1^Departamento de Neurobiología Conductual y Cognitiva, Instituto de Neurobiología, Universidad Nacional Autónoma de México, Querétaro, Mexico; ^2^Cuban Neuroscience Center, La Habana, Cuba; ^3^Centro de Investigación en Matemáticas, Guanajuato, Mexico; ^4^The Clinical Hospital of Chengdu Brain Science Institute, Ministry of Education, Key Laboratory for Neuroinformation, University of Electronic Science and Technology of China, Chengdu, China

**Keywords:** LD-NOS classification, EEG classification, stability based biomarkers, non-parametric ROC, sparse classifiers, elastic-net

## Abstract

In this paper, we present a novel methodology to solve the classification problem, based on sparse (data-driven) regressions, combined with techniques for ensuring stability, especially useful for high-dimensional datasets and small samples number. The sensitivity and specificity of the classifiers are assessed by a stable ROC procedure, which uses a non-parametric algorithm for estimating the area under the ROC curve. This method allows assessing the performance of the classification by the ROC technique, when more than two groups are involved in the classification problem, i.e., when the gold standard is not binary. We apply this methodology to the EEG spectral signatures to find biomarkers that allow discriminating between (and predicting pertinence to) different subgroups of children diagnosed as Not Otherwise Specified Learning Disabilities (LD-NOS) disorder. Children with LD-NOS have notable learning difficulties, which affect education but are not able to be put into some specific category as reading (Dyslexia), Mathematics (Dyscalculia), or Writing (Dysgraphia). By using the EEG spectra, we aim to identify EEG patterns that may be related to specific learning disabilities in an individual case. This could be useful to develop subject-based methods of therapy, based on information provided by the EEG. Here we study 85 LD-NOS children, divided in three subgroups previously selected by a clustering technique over the scores of cognitive tests. The classification equation produced stable marginal areas under the ROC of 0.71 for discrimination between Group 1 vs. Group 2; 0.91 for Group 1 vs. Group 3; and 0.75 for Group 2 vs. Group1. A discussion of the EEG characteristics of each group related to the cognitive scores is also presented.

## Introduction

Learning disability (LD), with a prevalence between 2.6 and 3.5%, (14% of all with mental health problems) in children between 5 and 16 years old (Emerson and Hatton, 2013), is a complex phenomenon that includes many facets. Definitions and classifications vary profoundly (Kavale and Forness, 1995). Usually a child is identified as suffering from LD when he/she has poor performance in standardized tests for reading, mathematics, and written expression–adjusted according to the age, schooling, and level of intelligence of the proband. Specific Learning Disorder are defined by problems in only one of these three areas: reading/language (Dyslexia), Mathematics (Dyscalculia), and Writing (Dysgraphia). On the other hand, there is another category, Not Otherwise Specified Learning Disability (LD-NOS) in which *more than* one area is affected (Diagnostic and Statistical Manual of Mental Disorders, version IV, DSM-IV-TR; American Psychiatric Association, 2000).

Thus LD-NOS is a broad, catch-all category for children with notable learning difficulties, which affect education but do not fall into a specific category. Obtaining a more nuanced assessment of these children may help to address subject-tailored therapy methods that better cope with their problems. However, due to the heterogeneity within and across domains it is challenging to disentangle the different possible subgroups of LD-NOS.

To our knowledge, subtyping of LD-NOS children has been only based on their behavioral and neuropsychological tests. Recently, Roca-Stappung et al. (2017) used cluster analysis based on the scores of the Neuropsychological Assessment of Children test (ENI) to find 3 clearly defined subtypes in a sample of 85 LD-NOS children. Children in Group 1 showed less severe problems; those in Group 2 showed an intermediate performance (without scoring very low in any of the tests); and group 3 had the most severe problems, significantly worse than the other groups in almost all tests. Thus, group membership is an ordinal scale variable that differentiates different levels of neuropsychological disabilities that may provide a key to the design of more specific rehabilitation. Nevertheless, further work in this direction might be substantially improved by the inclusion of neural biomarkers as a basis of stratification. A natural candidate for this type of biomarker are those derived from electrophysiology which is a non-invasive, inexpensive and sensitive technology for assessing brain dysfunction with relevance to low and middle-income countries.

The first stage for the identification of a biomarker is to demonstrate a significant variation of the selected features with the disease entity. The second is to verify their predictive power for retrospective studies. Progress for the first stage has been achieved for electrophysiological biomarkers of LD-NOS in work that can be summarized as below.

Event-Related Potential parameters have been shown by several authors to differ significantly between LD-NOS children and controls and between two subgroups of LD-NOS using this technique (Silva-Pereyra et al., 2001; Heine et al., 2013; Fernández et al., 2014; Tang et al., 2014; Žarić et al., 2014; Ma et al., 2016; Moll et al., 2016). Unfortunately, ERPs requires complicated experimental conditions and will not be the focus of our interest.

Quantitative Electroencephalography (qEEG) from the resting state is, on the contrary, a less difficult to apply method. qEEG parameters are clearly different between LD-NOS children and those with good academic achievement (Becker et al., 1987; Marosi et al., 1992, 1997; Fernández et al., 2002; Žarić et al., 2017). LD children are characterized by more power in the Theta band (4–7.5 Hz) and less amount of power in the range of alpha frequencies (8–13.5 Hz) (John et al., 1983; Lubar et al., 1985; Harmony et al., 1990; Marosi et al., 1992; Chabot et al., 2001; Fernández et al., 2002; Gasser et al., 2003; Fonseca et al., 2006). Even increases in power in the Delta band have also been observed in cases with severe difficulties (Harmony et al., 1990). Furthermore, Jäncke and Alahmadi (2016), showed significant qEEG differences between children with LD-NOS, those with learning disabilities with verbal disabilities (LD-Verbal), and healthy controls. The features were selected by using a group independent component analysis (gICA) model. Finally, in the study by Roca-Stappung et al. (2017) mentioned above it was shown that qEEG parameters differed between subtypes of LD-NOS.

In this paper, we turn attention to the second stage of qEEG biomarker identification for LD-NOS subtyping: that of the selection of a subset of parameters that have high predictive power. This is an old problem in multivariate statistics: variable selection for classification. The goal is to extract a small subset of relevant variables that can jointly classify subjects accurately into different populations. The solution of this task often becomes difficult in high dimensional settings, i.e., where there is a big number of variables involved in the problem and relatively small size of the sample (Mwangi et al., 2014; Jovic et al., 2015). Different approaches to variable selection have been used. The simplest one is to rank variables by means of standard univariate statistical methods such as the *t*-test and select those with significant scores. Its virtue is simplicity, but it entails a high number of individual tests that then require control of false positives for multiple comparisons. It also ignores the possibly important information contained in the correlations between variables. Multivariate discriminant analysis, on the other hand, does take advantage of the correlations when selecting variables for a discriminant function. The main problems with this approach is that, in high dimensional problems, there is a lack of stability in the selection of variables: different subset of biomarkers that exhibit similarly high classification accuracy on the training set then fail utterly in the test set. It is obvious that the training phase is capitalizing on chance.

Some methods have been introduced to circumvent the difficulties that arise for variable selection and classification in high dimensional settings (Meinshausen and Buhlmann, 2010; see Hastie et al., 2009; Fan and Lv, 2010 for comprehensive reviews). We base our own work on that proposed by Wehrens et al. (2011) who developed a method to achieve stability of potential biomarkers under perturbations. We further develop these ideas and apply them to the selection of biomarkers when there are several groups that are ordered in degree of severity as is the case for LD-NOS groups described by Roca-Stappung et al. (2017).

The aims of this paper are therefore two-fold:

- To improve a technique, first introduced by Wehrens et al. (2011), for the identification of stable classifiers on tests in high dimensional settings. This method will be extended to the prediction of disability severity
- To identify, using this technique, a stable low dimensional classifier, based on a minimal set of qEEG features, that predicts the degree of severity of LD-NOS.

## Sample and proposed qEEG feature set

### Participants

Eighty-five right-handed children (31 female) diagnosed with LD-NOS participated in the study. The age range was from 8 to 11 years (9.2 ± 0.96). They were tested with different scales: M.I.N.I.-KID (Sheehan et al., 2010), WISC-IV (Wechsler, 2007), and the Child Neuropsychological Assessment (ENI; Matute et al., 2007). They had normal neurologic examinations and no other psychiatric disorder. Their score at the Full-Scale Intelligence Quotient (FSIQ) was over 70, to exclude intellectual disability. The ENI evaluated three cognitive domains: reading, writing, and arithmetic. All subjects scored low in at least two of the three domains. A k-means cluster was applied to the ENI scores, dividing the sample into three groups. The number of clusters was previously decided by the experimentalists (Roca-Stappung et al., 2017).

The Ethics Committee of the Neurobiology Institute of the National Autonomous University of Mexico approved this study, which followed the Ethical Principles for Medical Research Involving Human Subjects established by the Declaration of Helsinki. Informed consent was signed by all children and their parents.

A detailed description of the experiment has been published elsewhere (Roca-Stappung et al., 2017).

### EEG recordings

The resting state EEG was obtained during rest, with the eyes closed, for the 19 leads of the 10–20 system, using the linked ear-lobes as reference. The data was sampled at 5 ms. To construct the EEG spectrum, for each subject, 24 artifact free segments of 2.56 s length were selected by an experienced neurophysiologist. This segment length is commonly used both in clinical and experimental EEG studies since it avoids non-stationarities in the EEG signal and guarantees an appropriate frequency resolution of 0.39 Hz for the analysis (Niedermeyer et al., 2010), with an accurate spectral description of the EEG signal, since the degrees of freedom of this estimate is larger than the number of electrodes. The EEG segments were re-referenced to the Average Reference. The data was then transformed to the frequency domain using the Fast Fourier Transform. The variables for the model were chosen from the standard high-resolution spectral model (Pascual-Marqui et al., 1988; Valdes-Sosa et al., 1990; Szava et al., 1993), which has been demonstrated to have a higher accuracy than the traditional broadband spectral models (Valdes-Sosa et al., 1990; Szava et al., 1993) since the summarization process involved in the calculation of the broad band models is a weaker approach for describing the EEG, since it can split a spectral peak between two bands (Szava et al., 1993). The spectra were calculated from 0.39 to 19.11 Hz yielding a parameter vector of 49 frequencies for each of the 19 leads, for a total of 931 variables. Spectra were rescaled by the Global Scale Factor (Hernández et al., 1994). An age correction was not applied to the data, since no significant differences with age were found among the groups. It must be noted that while the high-resolution spectral model has a higher dimensionality than the commonly used broad band estimation, we avoid, the danger of overfitting with the method described below.

## Statistical methods

The procedure to select a stable and sparse classifier is based on:

- The use of a sparse classifier based on the L1 penalty
- The evaluation of the performance of the classifier using Receiver Operator Characteristic (ROC) measures
- The use of resampling techniques to ensure variable selection that lead to stable classifiers.

We now describe each of these issues in turn.

### The sparse classifier: elastic net regression with prior screening

We carry out sparse classifier construction, with built-in variable selection, by estimating a weighted multivariate linear regression model known as the elastic-net (Zou and Hastie, 2005; Friedman et al., 2010). The model is described by the Equation (1):

(1) $$\begin{array}{l} \underset{\beta_{0}\in \mathbb{R},\;\beta_{0}\in {\mathbb{R}}^{p}}{\min}\left[\frac{1}{2N}\sum_{i=1}^{N}{\left(y_{i}-\varphi_{0}-x_{i}^{T}\varphi \right)}^{2}+\lambda P_{\gamma }\left(\varphi \right)\right] \end{array}$$

Here *N* is the number of subjects, ${\text{x}}_{i}\in {\mathbb{R}}^{p}$ observations of subject *i*, and **y**_*i*_ ∈ ℝ is the label group of subject *i*; φ_0_ ∈ ℝ, φ ∈ ℝ^*p*^ are the model parameters; γ is the regularization parameter; *p* is the number of variables in the model; and.

(2) $$\begin{array}{l} P_{\gamma }(\varphi )=(1-\gamma )\frac{1}{2}||\varphi |{|}_{l_{2}}^{2}+\gamma ||\varphi |{|}_{l_{1}} \end{array}$$

The penalty *P*_γ_ in Equation (2) is known as the elastic-net norm (Zou and Hastie, 2005). To understand its behavior, note that the ${||\varphi ||}_{l_{2}}^{2}$norm induces regressions that behaves well for high dimensional regressions but that tend to spread out coefficient weights among highly correlated variables. On the contrary, the ||φ||_l_1__ norm produces the “lasso regression” which is indifferent to highly-correlated predictors and tries to select only one thus inducing sparsity. The elastic-net reaches a compromise between the ridge and the lasso, the relative contributions being determined by the γ and λ parameters. Since these parameters are selected by cross-validation, in any specific case, the sparsity of the solution will be data-driven. The implementation of the elastic net described by Friedman et al. (2010) and Hastie et al. (2016), known as GLMNet, is implemented with high algorithmic efficiency by using cyclical coordinate descent methods. This fast implementation is essential to be able to carry out the iterative resampling techniques we use to achieve stability. We should also mention that GLMNet is able to cope with a wide family of models that includes not only the least square regression mentioned above, but also two-class logistic regression, and multinomial regression problems.

According to our experience, the GLMNet classification algorithm can deal well with problems with up to 1,000 variables, even when the number of subjects is less than 100. However, for the goal of dimensionality reduction, we applied an additional variable screening technique to eliminate variables with negligible contribution to the classification problem. This is the “indfeat” (Weiss and Indurkhya, 1998) “independent significance features test” (indfeat). For a single variable *X*, the “indfeat” index is the absolute value of a *t*-test for comparing group means. A feature is retained as a candidate if the significance value returned by the “indfeat” is larger than 1.0.

### Evaluation of the accuracy of classifiers

A widely used technique for assessing classifier performance is that of the receiver operator curve (ROC) methodology. Let us first consider the two-group classification scenario that attempts to distinguish between *D*^+^, the diseased (i.e., the positive condition) group ad *D*^−^ the healthy (i.e., the negative condition) group. Also suppose that there is a classifier that produces a continuous index *T* measured for both groups. The convention will be that higher values of test result are associated with greater severity of the disorder. We will classify a subject as positive if *T* ≥ *t* and this defines probabilities known as the specificity *Sp*(*t*) and the sensitivity *Se*(*t*)of the classifier:

$$\begin{array}{l} Sp\left(t\right)=P_{-}\left(T\leq t\right),\\ Se\left(t\right)=P_{+}\left(T\geq t\right) \end{array}$$

where *P*_−_ and *P*_+_ are, respectively the probability density of the index in both groups. The ROC curve is the plot of *P*_+_ vs. 1−*P*_−_(lack of specificity). A global measure of diagnostic accuracy is the Area under the ROC curve or (AUC). As can be easily seen AUC is 0.5 for a classifier that is operating at chance and is 1 for the perfect classifier. At a greater level of detail, to determine the threshold for which the detection level is optimal [the balance between *Sp*(*t*) and *Se*(*t*)], the Youden index was defined by Youden (1950) as:

$$\begin{array}{l} J\left(t\right)=Se\left(t\right)+Sp\left(t\right)-1 \end{array}$$

which reaches a maximum value of 1 if the test is perfect and a minimum of 0 when classification is at chance. An optimal threshold t^*^ can thus be obtained by taking $t^{*}\;=\arg\max_{t}J\left(t\right)$ and the optimal Youden index, which maximizes the overall effectiveness of a diagnostic test, will be the summary measure for its discriminatory ability.

We now generalize these concepts to the situation (as dealt with in this article) in which there are three ordered diagnostic groups based on the severity of a disorder or disease:

*D*^+^ (i.e., the positive condition) group.

*D*^0^ the intermediate group (early stage/very mildly diseased).

*D*^−^ the healthy (i.e., the negative condition) or, alternatively, the less affected group.

An AUC-like measure for multiple ordered groups was proposed by Obuchowski (2005). This author defines a non-parametric test that can estimate the marginal areas under the ROC between each pair of groups and the global area under the ROC for all the groups. The algorithm also allows the user to specify a parameter to penalize the effect of making a mistake in one specific sample, let us say to penalize the possibility of making a mistake with the positive class, i.e., the possibility of assigning an ill subject to the healthy population. The Youden index has also been generalized to the ordered three group scenario by Luo and Xiong (2013). Let *P*_*i*_ be the probability of the test in group, *D*^*i*^, *i* = +, 0, −. Now two threshold *t*_−_ and *t*_+_, with *t*_−_ <*t*_+_, are defined. Subjects whose T is below *t*_−_ are assigned to *D*^−^ and those above *t*_+_ to *D*^+^. The remaining subjects will be classified into the intermediate group *D*^0^.

The probabilities of correctly classifying patients from the three groups are individually defined as:

$$\begin{array}{l} Sp\left(t_{-}\right)=P_{-}\left(T\leq t_{-}\right),Specificity\\ Se\left(t_{+}\right)=\;P_{+}\left(T\geq t_{+}\right),Sensitivity\\ Sm\left(t_{-},t_{+}\right)=\;P_{0}\left(t_{-}\leq T\leq t_{+}\right),correctD^{0} \end{array}$$

The generalized Youden index for three groups is.

(3) $$\begin{array}{l} J\left(t_{-},t_{+}\right)=\frac{1}{2}\left[Sp\left(t_{-}\right)+Sm\left(t_{-},t_{+}\right)+Se\left(t_{+}\right)-1\right] \end{array}$$

*J*(*t*_−_, *t*_+_) allows the selection of optimal cutoff points as in the two-group case.

### Procedure for finding stable classifiers

With this framework in place, we now turn to the problem of finding stable classifiers, that is sets of variables, that have not been selected by chance. We follow here the central idea of Wherens, which is based on the idea of perturbations, that is to carry out variable selection on random subsamples obtained by resampling methods such as the jack-knife or bootstrap. If in any iteration some variables are selected by capitalizing on chance, it is very unlikely that they will be present in many other iterations of the method. More specifically, in each iteration, only the top 10% of variables (“top fraction”) is retained. The “top fraction” can be based either on the absolute values of either the *t*-values or the model coefficients. After all iterations, variables are ranked according to how frequently they were selected. Only variables that have a frequency of selection above a “consistency threshold” are retained as useful biomarkers. Wehrens et al. (2011) used the AUC to pick the “consistency threshold”—which is recommended to be 50%.

In this paper, we extend the methodology proposed by Wehrens et al. (2011) in several directions:

- Instead of selecting the “top fraction” of variables to be retained for each iteration we carry out this selection based on a doubly sparse procedure: preselection of variables, first by the indfeat feature selection procedure and then subsequent further sparsification by means of the elastic net regression.
- The standard AUC estimation procedure is sensitive to small perturbations in the sample, especially when the number of variables exceeds the sample size (*p*>>*N*) (Pencina et al., 2008; Gu and Pepe, 2009). To solve this problem, we implement here a stable estimate based on the empirical statistical distribution of the ROC areas, by further random samplings of the data. The value at the 50% of the distribution is adopted as a stable estimator of the AUC.
- For the estimation of the ROC curve we use a non-parametric method (Obuchowski et al., 2001; Obuchowski, 2005, 2006) valid when the gold standard is not binary, i.e., continuous, ordinal o nominal scale as in our case. The method allows assessing the discrimination accuracy by calculating the marginal area under de ROC between each pair of groups. The generalization of the ROC to multi-class has received much attention by researchers in the last decade (Nakas and Yiannoutsos, 2004).
- Additionally, to give a numerical index of the specificity and sensitivity of the method, we used the Youden index for the ordered three-group data described in the previous section.

### Summary of the method for stable classifier

**Figure 1:**
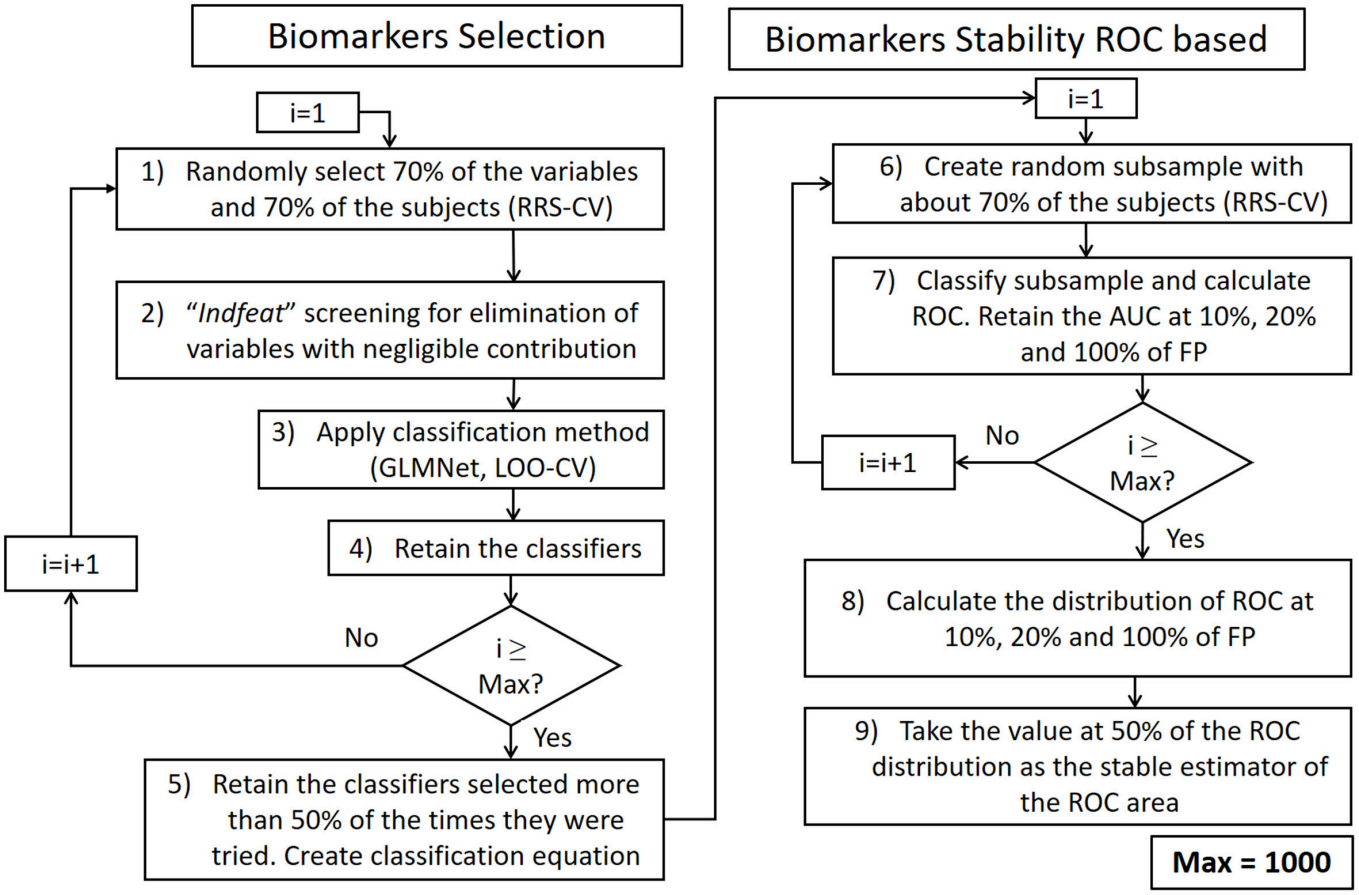
Schematic representation of the Robust Sparse classification algorithm with stable ROC assessment. LOO-CV, Leave-One-out cross-validation; RRS-CV, Repeated Random Subsampling cross-validation.

Our procedure for the identification of stable classifiers is shown in Figure 1 in a schematic form. It consists of two parts:

#### Biomarker selection (left panel of Figure 1)

1. For several iterations *i*, the algorithm selects a random subsample that includes 70% of the original individuals and 70% of the variables.
2. Indfeat screening with a threshold of 1.0 is used to prune the total variable set.
3. The GLMNet procedure is then applied to obtain a minimal set of variables for the iteration *i*. the variables selected at this iteration are kept for further analysis.
4. After a number of Max_Iterations (1,000 in our case), only the variables that are selected more than 50% of the times will be in the final selection.
5. The final classifier is found using GLMNet with the selected variables.

Note that our algorithm uses two different cross-validation procedures: the first one is the Leave-One-Out cross-validation (LOO-CV), included in the selection of the regularization parameters of the GLMNet algorithm; the second one is the subsampling of the variables and subjects in the step 2) of the loop in each iteration. This type of cross-validation is known as Repeated Random Subsampling or Monte Carlo cross-validation (RRS-CV) (Dubitzky et al., 2007, Chapter 8), which is more restrictive than the commonly used leave-one-out technique and it is one of the strongest cross-validation procedures possible.

#### ROC based biomarkers stability assessment (right panel of Figure 1)

6) For a given iteration *i*, a random a subsample of 70% of the original individuals the variables are formed.
7) With the remaining 30% of the subjects the Area under the ROC (AUC) curve is calculated.
8) After a number of Max_Iterations (1,000 in our case), the distribution of the AUC is found.
9) The AUC at the 50% of the distribution is retained as the measure of classification accuracy.

Note that also in this phase we use RRS-CV in step 6) during the construction and validation of the robust ROC curves.

## Results

### Variable selection

The methodology described in the previous section was applied to the 931 variables of the EEG spectra of the 85 LD-NOS children, divided into the three groups with increasing disability.

As a first step, the “indfeat” index was calculated between each pair of groups of the sample for all variables in the model. The variables that did not exceed the significance level of 1.0 for any pair of groups were discarded as candidate predictors. Because of this procedure, 364 variables were removed. The “biomarkers selection” step (see Figure 1 left panel) was applied to the remaining 537 variables, which identified 20 variables that were selected as biomarkers.

Table 1 shows the results of the classification procedure. The Table has been sorted by the frequencies in Hz, to facilitate comparison to the traditional Broad Band definition of EEG frequencies. Columns 1 and 2 show the Lead and frequency selected as biomarkers. Column 3 shows the proportion of times each variable appears over iterations. Column 4 shows the coefficients φ with which each variable was included in the final classification equation. Finally, Column 5 groups the selections according to the Broad Band model.

**Table 1:** Selected biomarkers during the classification step.

**Lead**	**Frequency**	**Percent**	**Coeffcients φ**	**Broad Band**
C4	0.39	86.5	0.12	
T6	0.39	58.89	0.12	Delta
T4	1.17	59.46	0.07	
P4	3.52	59.55	−0.18	Low Theta
P4	3.91	61.4	−0.3	
P3	5.08	69.6	0.23	
F8	5.08	57.5	0.16	
Fp1	5.08	54.3	0.06	
Fp2	5.47	58.6	0.03	High Theta
Fp1	5.47	57.5	0.06	
Fp2	5.86	56.5	0.03	
Fz	6.64	69.8	0.16	
C3	7.81	60.8	−0.11	Alpha
T6	8.59	54.5	−0.11	
T6	10.16	56.44	−0.04	
C3	10.94	53.7	−0.16	
P3	14.06	56.7	0.21	Beta
P3	15.23	62.3	0.34	
F3	15.63	57	−0.25	
O1	18.36	52	0.27	

*Columns 1 and 2 contain the Lead name and the selected frequency; Column 3 contains the percent of times that the variable was identified as a biomarker. In Column 4 the Beta coefficients for each variable in the classification equation*.

**Figure 2:**
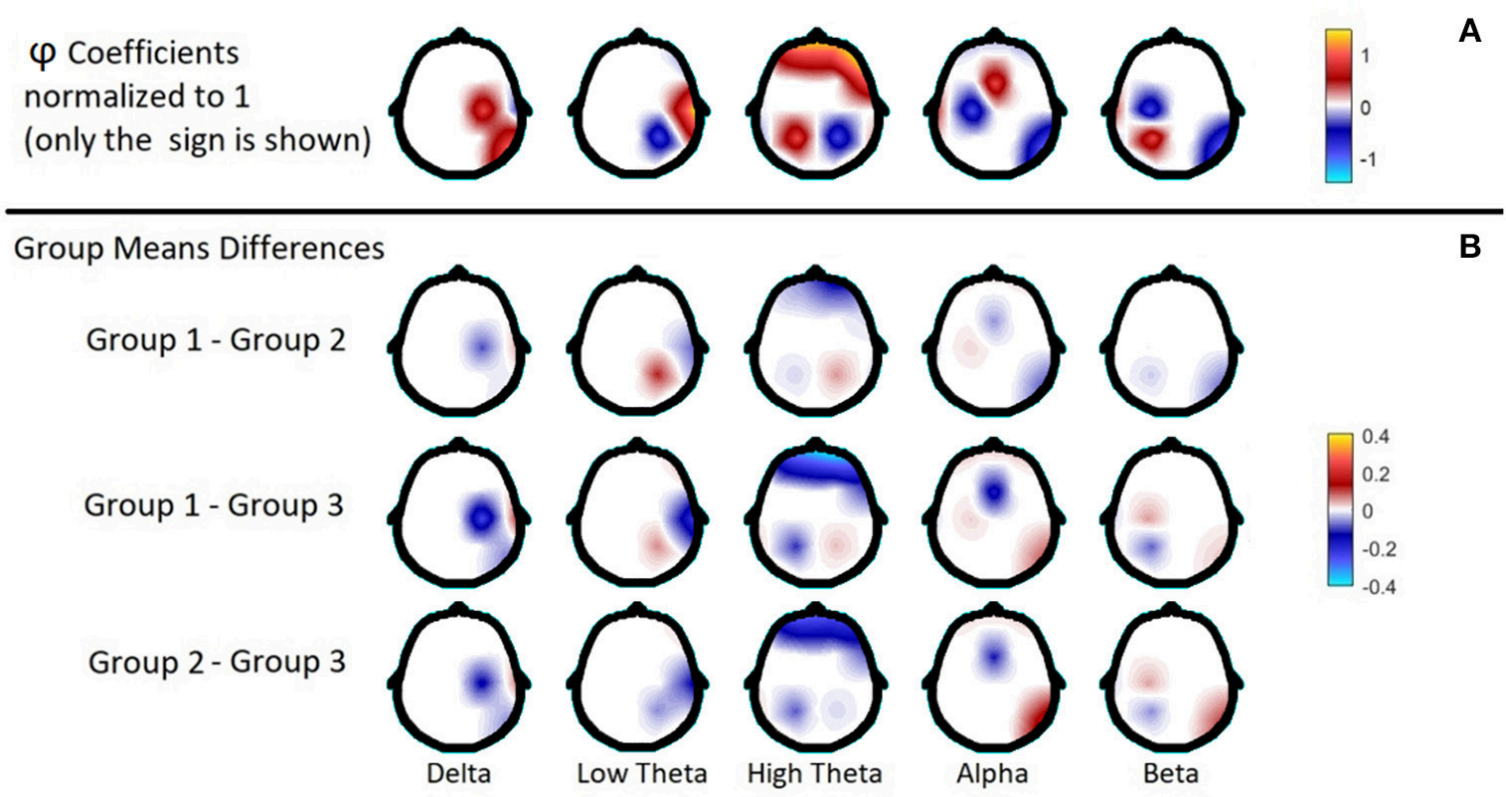
Color-plate with the φ coefficients of the classification equation **(A)** and the differences of the mean EEG spectra between each pair of groups, at the selected leads by the biomarkers procedure **(B)**. Everything has been summarized by the Broad Bands shown in Table 1. The φ coefficients have been normalized to show only the sign of the coefficient.

Figure 2 summarizes the distribution of the biomarkers both in topography as well as frequency. The first row of Figure 2 shows the topographical distribution of the φ coefficients, summarized by the traditional broadband frequencies, to comparisons. Note that due to the sparse nature of the biomarkers technique, there are not wide areas selected as biomarkers, as it is commonly seen in classification techniques based on statistical thresholds. The elastic-net technique used here tries to avoid selecting variables as biomarkers which contain approximately equivalent information, which is the case of adjacent frequencies and electrodes. Note however, that the biomarkers are not randomly distributed. For example, in Delta there are three adjacent electrodes in the right hemisphere (C4, T4, and T6); in High Theta there is a wide frontal area; in Beta the variables are in the parieto-occipital areas (P3 and O1) and one in the frontal area (F3) of the left hemisphere. It is also important to observe the colors in the figure. The colors indicate that the variables have the same sign in the classification equation (i.e., same effect) and the colors are well-organized by brain regions.

Rows 2 to 4 in Figure 2 show the average differences of the mean values of the spectra of each pair of groups in each frequency band.

Additionally, to facilitate the analysis of the results, Table 2 shows a summary of the clinical and the electroencephalographic findings for each group. EEG findings are extracted from the mean differences between the groups shown in Figure 2 and the cognitive findings in Roca-Stappung et al. (2017).

**Table 2:** A comparison between the EEG patterns and the Cognitive findings for each group.

**Group**	**EEG findings**	**Cognitive findings**
Group 1	Highest Low-Theta in P4 Highest Alpha in C3 Highest Beta in F3, P3	Highest scores in Reading (Accuracy, Comprehension, and Speed); Writing Accuracy and Arithmetic Calculation and Numeric Management. Significantly best in Reading and Writing Accuracy.
Group 2	Highest Alpha in T6	Highest scores in Writing Narrative Composition; and Arithmetic Counting. Significantly best in Writing Narrative Composition.
Group 3	Highest Delta in C4 and T6 Highest High-Theta in Fp1, Fp2, Fz, P3, and P8 Highest Beta in P3, O1	Poorest scores in all areas, especially in Arithmetic (Calculation and Numeric Management); Writing Narrative Composition; and Reading Accuracy.

### Performance of the classifier

The stable marginal AUC of the ROC for each pair of groups are shown in Table 3. There is a high discrimination power of the classification equation for Groups 1 and 3 (0.91). The value of the marginal AUC for Groups 1 and 2 and for Groups 2 and 3 also exhibit a good classification accuracy over 0.7.

**Table 3:** Marginal and global AUC after the stability based procedure for ROC estimation.

**AUC**			
**Group 1 vs. Group 2**	**Group 1 vs. Group 3**	**Group 2 vs. Group 3**	**Global**
0.71	0.91	0.75	0.89

**Figure 3:**
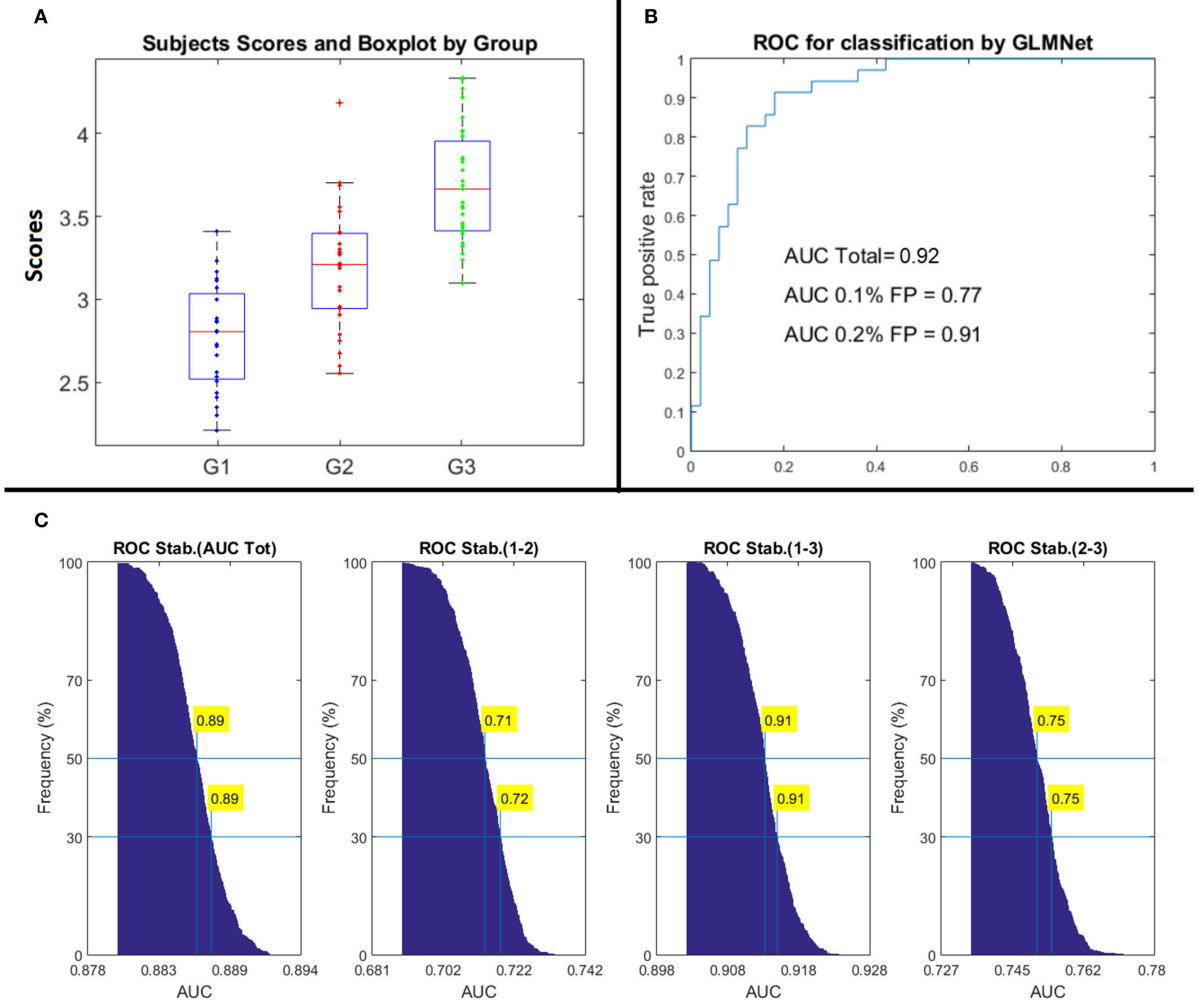
Performance of the classification method applied to the 85 LD-NOS children **(A)** is a boxplot of the individual classification according to the groups. As defined, the boxplot shows the mean, percentiles, and dispersion of the groups. Note that Group 3 is almost perfectly separated from Groups 1 and 2 **(B)** shows the ROC curve for the global performance of the algorithm, before applying the ROC stability procedure. The rate of True Positive at a rate of 10 and 20 percents of False Positive is very high **(C)** shows the performance of the ROC curve under the stability procedure. Note the stable ROC estimate for the Global classification as well as the Marginal estimates for each pair of groups.

Figure 3A shows the scores obtained for each subject in each group evaluated with the classification equation. A boxplot is included to show the mean and quantiles of each group. The third group is well-separated from the first two groups, explaining the large AUC shown in Table 3. The right panel of Figure 3, shows the global ROC curve for the classification equation. Note that the total area under the ROC curve is 0.92, which is higher than the area reported in Table 3 (0.89) since it has not been yet submitted to the stability procedure described in the right panel of Figure 1, which yields a more conservative estimate. Note also that the value of the ROC curve at 10% of False Positive is 0.77 and the value at 20% of False Positive is 0.91, which means that at a low rate of False Positives the sensitivity of the equation is high, a very desirable property.

Panel C in Figure 3 shows the results of obtained with the stability procedure of the ROC, after *Max_iterations* = 1,000. The figures show the empirical distribution of the AUC for the global area and the pairwise areas after all iterations. In each iteration, the nominal ROC is obtained by selecting a random subsample of the original sample. The values for the total AUC as well as for each pair of groups are stored. At the end, the stable estimation of the AUCs is selected as the value at the 50th percentiles of the empirical distributions (see Figure 3C).

### Optimal sensitivity and specificity of the model (Youden index)

The Youden index described in section Evaluation of the Accuracy of Classifiers was calculated for the stable classification equation. Table 4 shows the summary of the data basic statistics produced by the method. The Youden index for this model was **0.4838**. The Confidence Interval (CI) at the 95% of variance was **[0.38 0.59]**.

**Table 4:** Youden Index.

**Group**	**N**	**μ**	**σ**
D− (Group1)	24	2.77	0.32
D0 (Group2)	26	3.19	0.38
D+ (Group3)	35	3.68	0.33

Raw Data Summary

The best cut-points found by the Youndex index in this case are: lower = **3.01**, upper = **3.42**. These values allow the Youden index not only to summarize the discriminatory accuracy of the diagnostic test but also to provide a ready-to-use optimal cut-point for future diagnosis. Table 5 shows the group correct classification probabilities, for the selected cut-points.

**Table 5:** Group correct classification probabilities, for the best Youden cut-points.

**Specificity (Sp)**	**Correct classification probability (Sm)**	**Sensitivity (Se)**
0.77	0.41	0.79

### Comparison of the results with those of random samples and other classification methods

**Figure 4:**
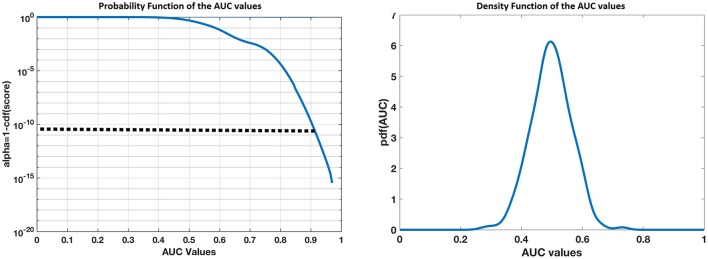
Distribution of the AUC values obtained by 1000 random realizations of the classification algorithm. **Left** shows the probability function of the AUC in the range 0 to 1 and the **right** shows their density distribution function. Note that the probability of obtaining by chance an AUC value of 0.91 (like ours) is smaller than 0.1e-10, which is in practice an impossible event. Also, the density distribution is centered at 0.5 (random classification), which coincides with the mean value of the AUC in our random realizations.

To explore whether results just described (see Table 2) are not produced by chance (at random), we carried a further 1,000 realizations of our complete classification procedure, reordering group membership of the random in each iteration. For each iteration, we calculated the total area under the ROC curve. The distribution of those ROC values is at the chance level (mean = 0.497). To statistically assess this result, we took the distribution of the AUC values and calculated their density distribution as well as the probability of the density function in the range 0 to 1. These results are shown in Figure 4. The left panel in Figure 4 shows the probability function of the AUC. Note that the probability of obtaining by chance an AUC value of 0.91 (like ours) is smaller than 0.1e-10, which is in practice an impossible event. The right panel of Figure 4 shows the density distribution of the values of the AUC at random level. Note that it is centered at 0.5 (random classification), which coincides with the mean value of the AUC in our random realizations.

Finally, to compare the performance of our method with other standard methods used in the literature, we used two classification algorithms included in Matlab R2015a:

- A multiclass model for the Support Vector Machine (SVM) algorithm (Allwein et al., 2000; Fürnkranz, 2002; Escalera et al., 2009) (function named “fitcecoc” in Matlab).
- A regularized linear discriminant analysis (LDA) classifier (Guo et al., 2007) (function name in Matlab “fitcdiscr” with Linear discriminant).

For both algorithms several leave-one out repetitions were carried out by successively leaving one subject out of the classification procedure and then evaluating it with the obtained classification equation as a totally independent testing sample. We thus obtained an unbiased estimate for the ROC curve. The resulting area under the ROC curve by the LDA algorithm was **0.58**; and the area under the ROC for the SVM procedure was **0.65**, both significantly smaller than the **0.91** obtained with our methodology.

## Discussion

In this paper we report a method to achieve stable classifiers for qEEG parameters, with two main objectives, to improve a technique for the identification of stable classifiers, extending this to the prediction of disability severity and the identification using this technique of a minimal set of qEEG features (biomarkers) to predict the degree of severity of LD-NOS.

**A) To improve a technique (Wehrens et al., 2011), for the identification of stable classifiers on tests in high dimensional settings**.

Our approach is consistent with best practices in bioinformatics and neuroinformatics where there are many more variables than subjects. To avoid capitalizing on chance, the neuroimage community has recommended the use of resampling techniques as described in the special issue of Neuroimage “Individual Subject Prediction” (Arbabshirani et al., 2017; Stephan et al., 2017; Varoquaux et al., 2017). We emphasize that our method adheres to these procedures using the elastic-net technique, which includes a regularization parameter to shrink the number of variables which will participate in the model. This is a common and effective technique to avoid overfitting since the model tries to reduce the number of parameters in a natural data-driven way. Additionally, the estimation of the regularization parameter (lambda) is performed by means of cross-validation.

The stability based ROC procedure evidenced high sensibility of the classification equation to discriminate between the groups, especially Group 3. This result points to different EEG patterns for each group, which may be an evidence of the different neurological origin of the learning disabilities, although all children have been classified in a very wide unspecific group. The current classification of LD-NOS may not be appropriate for the best understanding of the characteristic and needs of these children. Finding specific EEG alterations in each group may lead to a better classification of the children affected by this disorder, which may also be useful for the design and development of subject-tailored rehabilitation methods.

**B) To identify, using this technique, a stable low dimensional classifier, based on a minimal set of qEEG features (Biomarkers), that predicts the degree of severity of LD-NOS**.

We identified a set of 20 qEEG features (see Table 1) which allowed the classification of the groups. The Group 3 obtained the lowest scores in the three cognitive areas (see Table 3); their scores were extremely low compared to Groups 1 and 2. Groups 1 and 2 were more balanced; although children of the Group 1 performed better in most tests, they were especially good in Reading and Writing accuracy; the Group 2 performed significantly better in Writing Narrative Composition.

For the description of the classifiers we will use the EEG basic rhythms and/or band of frequency.

### Theta band

Theta band has been divided into Low and High Theta for convenience due to the topographical distribution of the classifiers. In the Low Theta, only the right parietal area (P4) was selected, and Group 1 had the highest values. In the High Theta, bilateral frontal areas (Fp1, Fp2, F8, and Fz) and the left parietal (P3) areas were involved, where Group 3 had the highest activity. The excess of Theta activity in the EEG at rest has been consistently reported in LD-NOS children (Mechelse et al., 1975; Colon et al., 1979; John et al., 1983; Harmony et al., 1990; Alvarez et al., 1992; Fernández et al., 2002; Jäncke and Alahmadi, 2016). This seems to be consistent with the fact that Group 3 obtained the lowest scores and, therefore, should be more affected. Compared with children with good academic Achievements, LD-NOS children had evidenced excess of Theta activity (from 3.52 to 7.02 Hz) (Fernández et al., 2002). However, some authors who have studied this entity have not reported excess in Theta activity (Byring et al., 1991; Chabot et al., 2001; Gasser et al., 2003; Fonseca et al., 2006; Thatcher et al., unpublished manuscript), although it may be due to the composition of their samples and to the frequency of different types of pathological patterns in the extensive group of LD-NOS.

It is also interesting to note the presence of frontal and parietal electrodes in the classifiers. Even if the electrodes reflected only electrical activity at the scalp, a common practice is to match this activity with the corresponding brain structures. In that case, we can say that frontal and parietal lobules are involved in the attention processes: (a) the dorsal network of attention in charge of the spatial orientation, involved frontal and parietal areas; (b) the ventral network of attention, in charge of the detection of the environmental stimuli, involved the temporoparietal joint and the ventral frontal cortex, mainly in the right hemisphere. This corresponds to the parietal and frontal cores of the orienting function (Petersen and Posner, 2012). Although LD-NOS children do not satisfy the DSM-IV criteria for Attention Deficit Disorder and Hyperactivity (ADDH), they frequently have from mild to moderate attentional deficits.

The frontal regions also process the executive functions as inhibition processes, planning, and working memory. Swanson (1987) proposed that the main deficit of LD children lies in mechanisms of executive functioning, which also points to working memory deficits as essential problems in children and adults with LD (Swanson and Siegel, 2001; Berninger, 2008), specifically in Baddeley's proposed phonological loop and central executive (Fletcher, 1985; Landerl et al., 2009; Maehler and Schuchardt, 2011; Swanson, 2012; Swanson and Stomel, 2012).

### Delta band

The presence of biomarkers at 0.39 Hz is somehow unexpected, but it was carefully tested. This very slow frequency is usually associated with ocular movements if it appears at frontal leads, but in this case, it appeared at the right central-parietal leads. (Steriade and Timofeev, 2003) hypothesized that frequencies below 1 Hz are not Delta rhythm properly but slow oscillations which, to some extent, modulates the Delta rhythm.

### Alpha band

The presence of posterior (T6) alpha rhythm has been related to the maturational process (John et al., 1983; Harmony et al., 1995; Riviello et al., 2011) and the number of correct answers in experimental conditions (Klimesch, 1999). The alpha biomarker in the left central lead (C3) may correspond to the sensorimotor rhythm (SMR). The reinforcement of SMR has been successfully used in Neurofeedback in the treatment of epilepsy (Sterman and Egner, 2006) and attention deficit disorder / hyperactivity (ADHD; Monastra et al., 2005). Pineda (2005) has found this activity related to cognitive performance. Group 1 and Group 2 exhibited the highest alpha values, which seems to be consistent with the hypothesis that they have a more matured brain than the children in Group 3.

### Beta band

Children of Group 3 have higher Beta power than the other two groups in frontopolar, anterior temporal and left parietal electrodes. Several studies have found the existence of one group of children with the combined type of ADHD which have an EEG profile characterized by excess of Beta activity (Chabot et al., 2001; Clarke et al., 2001a,b,c). We already pointed out that although LD-NOS children of our study might have attentional problems, they do not meet the criteria to be diagnosed with ADHD; therefore, it is possible that children in the Group 3 were distinguished by having more attentional problems than those of the other two groups, that may explain the greater difficulties observed in children of the Group 3. On the other hand, the temporal and left parietal regions are involved in language and calculation processes, in which these children have lower performance than other children with LD.

In our study the selected biomarkers agree with previous studies distinguishing LD from normal children (Colon et al., 1979; John et al., 1983; Harmony et al., 1990; Alvarez et al., 1992; Fernández et al., 2002; Jäncke and Alahmadi, 2016; Žarić et al., 2017). Most of the biomarkers were found in the Theta band as in previous studies, and the biomarkers in the Delta and Alpha bands have also been described to discriminate between these two groups. However, our new approach found that in each of the groups classified according to these biomarkers it is possible to distinguish also different behavioral characteristics between groups. These results are extremely valuable. Since the practical point of view, the EEG may guide the type of rehab-educational therapy, paying special attention to those with the EEG characteristics of the group 3, since these children showed the worst performance in the neuropsychological tests.

In summary, this is the first report using quantitative EEG to try to obtain subtypes within the group of children with LD-NOS abnormalities.

This is more relevant because the LD-NOS constitute a very heterogeneous group. For this reason, to apply a new procedure of analysis of the EEG to classify according to their electrophysiological characteristics (that represent the bases of behavior) is a step forward to understand their differences to explore specific new therapeutic procedures.

In the future, research using joint recordings of EEG-fMRI, in resting state or during task-related paradigms, can be a more complete validation of the biomarkers already found here, taking advantage of the MRI spatial resolution. This could shed more light about the brain structures related to each subtype.

Note that the “indfeat” procedure is an optional step inside the classification algorithm to eliminate non-informative variables. It can be avoided, or it can be performed just once before the classification. In fact, there exist other algorithms to reduce dimensionality that might be used instead. We also tested the algorithm using “indfeat” just once, outside the main loop, and obtained essentially the same results (not shown but available upon request to the authors).

## Conclusions

We have shown that resampling techniques adequately protect against the curse of dimensionality when constructing classifiers from high-dimensional, small size samples. We extend the methodology by Wehrens et al. (2011), for the identification of stable classifiers for predicting degree of severity.

We apply this methodology to find an optimal predictor of LD-NOS disability severity based on a reduced set of qEEG variables that may be of use in real world screening settings.

The selection of a small set of qEEG variables with good predictive properties is of importance in practice since it would allow designing stripped-down EEG devices that could be cost/effective and deployable in all economic settings.

## Author contributions

JB-B participated in methods development and programming, data analysis, and processing, manuscript writing, discussions, hypothesis elaboration, figures creation; LG-G participated in methods development, programming, discussions, manuscript revision, hypothesis elaboration; TF participated in the experiment design, data collection, manuscript revision, discussions, hypothesis elaboration; RL participated in methods development, manuscript writing, discussions, hypothesis elaboration; MR-S participated in the experiment design, data collection, and hypothesis elaboration; JR-G participated in EEG analysis, discussions, and hypothesis elaboration; MB-V introduced new methods for sensitivity and specificity and processed data in R; TH participated in discussions, hypothesis elaboration, manuscript revision; PV-S participated in the mathematical formulation, programming, discussions of the methodology, methods advisor, hypothesis elaboration, and writing of the article.

### Conflict of interest statement

The authors declare that the research was conducted in the absence of any commercial or financial relationships that could be construed as a potential conflict of interest.
